# A 3D Printed Implantable Device for Voiding the Bladder Using Shape Memory Alloy (SMA) Actuators

**DOI:** 10.1002/advs.201700143

**Published:** 2017-07-26

**Authors:** Faezeh Arab Hassani, Wendy Yen Xian Peh, Gil Gerald Lasam Gammad, Roshini Priya Mogan, Tze Kiat Ng, Tricia Li Chuen Kuo, Lay Guat Ng, Percy Luu, Shih‐Cheng Yen, Chengkuo Lee

**Affiliations:** ^1^ Department of Electrical and Computer Engineering Faculty of Engineering National University of Singapore 4 Engineering Drive 3, #05‐45 Singapore 117583 Singapore; ^2^ Singapore Institute for Neurotechnology National University of Singapore 28 Medical Dr. #05‐COR Singapore 117456 Singapore; ^3^ Center for Intelligent Sensors and MEMS National University of Singapore 4 Engineering Drive 3 Singapore 117576 Singapore; ^4^ Biomedical Institute for Global Health Research and Technology (BIGHEART) Yong Loo Lin School of Medicine National University of Singapore 14 Medical Drive #14‐01 Singapore 117599 Singapore; ^5^ Raffles Hospital 585 North Bridge Road Singapore 188770 Singapore; ^6^ Singapore General Hospital Outram Road Singapore 169608 Singapore

**Keywords:** 3D printing, actuators, flexible electronics, shape memory alloy, under active bladder

## Abstract

Underactive bladder or detrusor underactivity (DU) is defined as a reduction of contraction strength or duration of the bladder wall. Despite the serious healthcare implications of DU, there are limited solutions for affected individuals. A flexible 3D printed implantable device driven by shape memory alloys (SMA) actuators is presented here for the first time to physically contract the bladder to restore voluntary control of the bladder for individuals suffering from DU. This approach is used initially in benchtop experiments with a rubber balloon acting as a model for the rat bladder to verify its potential for voiding, and that the operating temperatures are safe for the eventual implantation of the device in a rat. The device is then implanted and tested on an anesthetized rat, and a voiding volume of more than 8% is successfully achieved for the SMA‐based device without any surgical intervention or drug injection to relax the external sphincter.

## 1 Introduction

Voluntary urination requires the contraction of the detrusor muscle and simultaneous relaxation of the sphincter muscle. This is usually facilitated by neural signals from the brain that are relayed through the spinal cord.^1^ The bladder contractions and the status on bladder filling is controlled by parasympathetic nervous system, while the permission for volitional micturition is granted by the somatic efferent system.^1^ Prolonged urination, often with altered sensation, impaired sense of emptying, and a slow stream, are symptoms that are characteristic of an underactive bladder (UAB).^2^, ^3^ Incomplete emptying of the bladder may happen due to the atrophy of the detrusor muscle, i.e., myogenic UAB or detrusor underactivity (DU), or disease or damage to the afferent system, i.e., neurogenic UAB, and can lead to overflow incontinence.^4^ The inability of the detrusor muscles to empty the bladder, which is the clinical motivation for this paper, may happen as a result of aging, undesired side effect of pelvic surgery, or neurological disorders (i.e., Parkinson's disease, multiple sclerosis, metabolic disorders such as diabetes, etc.).^4^ It is a widespread condition, ranging from 9% to 23% in men younger than 50 years old, increasing to as much as 48% in men over 70 years.^5^ Among elderly women, prevalence ranges from 12% to 45% have been reported.^5^

A limited number of treatments have been reported for restoring the control of the bladder.^6^, ^7^, ^8^, ^9^ The use of available oral drugs show limited clinical benefits,^10^, ^11^ and have numerous undesired side effects.^12^ Willing and physically able patients can use intermittent self‐catheterization. The disadvantages of intermittent catheterization include occurrence of infection, possibility of pain experienced during catheter insertion, and requirement for the catheterization several times a day. For patients with insufficient manual dexterity, a prohibitive body habitus, or a psychological lack of acceptance, indwelling catheterization can be considered.^1^ The intraurethral valve‐pump is a nonsurgical urinary treatment to initiate bladder drainage for women with DU.^1^ The valve‐pump consists of a miniature internal pump that inserted through the urethra, and the patient can magnetically activate the pump. Discomfort and leakage of urine are the drawbacks of using this device.^1^ Latissimus Dorsi detrusor myoplasty is another alternative treatment, which requires a major surgical procedure.^13^ The other alternative treatment includes neuromodulation techniques.^14^, ^15^ Current neurostimulation methods to obtain bladder voiding are more reversible than drugs, and avoid systemic effects due to a confined nerve target, but are not without shortcomings. For example, approved Food and Drug Administration (FDA) bladder neuromodulation devices stimulate either the sacral nerve roots or the pudendal nerves, in which the bladder contraction is achieved with off‐target activation of other neural pathways or by indirectly triggering the micturition spinal reflex.^1^ In addition, these nerve stimulation devices will not work in patients with injured or damaged sacral or pudendal nerves, or patients with degenerated detrusor muscles.

In contrast to current treatments, we propose to build a flexible implantable actuating device to physically and directly contract the bladder on demand in order to achieve facilitated micturition for UAB and avoid urine retention. The implantation of our proposed device requires a minor surgical procedure as it will be fitted once onto the bladder surface and used continuously for life, since the actuating part of the device (i.e., shape memory alloy (SMA) wires) is capable of repeated deformations on the order of tens of millions of cycles.^16^ Since the urethral resistance, especially due to the movement of pelvic floor muscles plays an important role in the micturition as well as the contractility force of the detrusor,^17^ therapies aimed at lessening urethral resistance would also be advisable while implanting the device.^1^

There has been increasing interest in flexible electronics for implantable devices as they can often conform very well to biological tissues (i.e., brain, heart, nerves, etc.).^18^, ^19^, ^20^, ^21^, ^22^ On the other hand, SMA actuators are of interest for medical rehabilitation particularly due to their material compatibility, small weight, and capability for voluntary movement.^23^, ^24^, ^25^, ^26^, ^27^, ^28^ Usage of SMA actuators for muscular contraction to overcome the lost or impaired motor function has been reported previously.^26^, ^27^, ^28^ However, this is the first time that these wires are used for physical contraction of the bladder with the help of a flexible,^29^ 3D printed vest. Moreover, the integration of deformable thin SMA wires on a flexible vest will help the surgeon to easily compress the device for the implantation inside body through a small incision and let it expand to the original size inside the body.

3D printing technology is becoming an attractive manufacturing field for rapid prototyping of biomedical structures with the geometrical details close to the original biological organ.^30^ The dimensions of the 3D vest for each patient can be customized based on images taken with common imaging techniques (e.g., ultrasound) for the bladder.^31^

Various designs of the proposed device were tested by using an experimental setup to mimic the bladder. This was followed by an in vivo test of the device in an animal. The design and fabrication of the device is first presented, followed by numerical modeling of the forces exerted by SMA wires of different lengths on different designs of the 3D printed vest. After that, the experimental setup for the characterization of the devices using balloon as a model of the bladder is discussed. The results, including the percentage of water voided, operational temperatures, and hysteresis behavior of the SMA‐based devices are presented in the following sections. The optimum device design with the highest contraction force while maintaining lowest operational temperature, was then implanted in a rat model.

## 2 The SMA‐Based Device for Myogenic UAB

**Figure 1:**
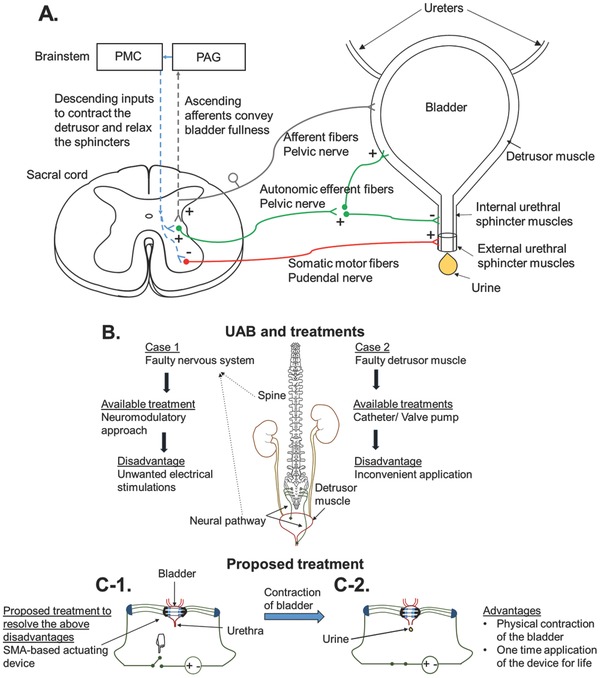
A) The neural pathways and muscles involved in micturition (plus signs (+) indicate excitatory synapses while minus signs (−) indicate inhibitory synapses). Inhibition of the sympathetic pathway during micturition is not shown for simplicity. B) The causes of UAB and current treatments. C‐1,C‐2) The proposed treatment and its operation.

**Figure** **1**A illustrates the neural pathways and muscles involved in micturition. The stretch receptors in the bladder wall sense bladder fullness, and convey signals via the afferent fibers of the pelvic nerve toward the spinal cord and the brainstem.^32^, ^33^, ^34^ The periaqueductal gray and pontine micturition center regions in the brainstem integrate the afferent signals, and upon bladder fullness, initiate the micturition process by sending down descending inputs to the sacral spinal cord to contract the bladder detrusor muscles and relax the sphincter muscles. Excitation of the autonomic efferent fibers of the pelvic nerve by the descending inputs leads to contraction of the detrusor muscle and relaxation of the internal urethral sphincter required for voiding. Inhibition of the somatic pudendal pathway by the descending inputs results in relaxation of the external urethra sphincter muscles.^32^, ^33^, ^34^

The causes for UAB and the current treatments and their drawbacks for UAB patients are summarized in Figure 1B. The proposed device in this work is to be used for voluntary voiding of the bladder upon sensation of fullness, and can be used by at least two types of patients: (1) myogenic patients with intact nerve function but degraded muscle function; and (2) neurogenic patients with degraded nerve function but intact muscle function. For myogenic UAB patients with intact nerve function, they may still be able to rely on the natural pathways to realize when their bladder is full.^35^ However, for neurogenic patients, a sensor may have to be used to alert the patients when their bladder is full.^36^, ^37^ The proposed SMA‐based device in this paper can be an alternative treatment for both myogenic and neurogenic UAB patients. However, our focus in this paper is on myogenic UAB. Figure 1C shows the schematic contraction of the SMA‐based device, and the consequent voiding of the bladder after activating the device. The preparation of the device and the related numerical modeling are reported in the following sections.

**Figure 2:**
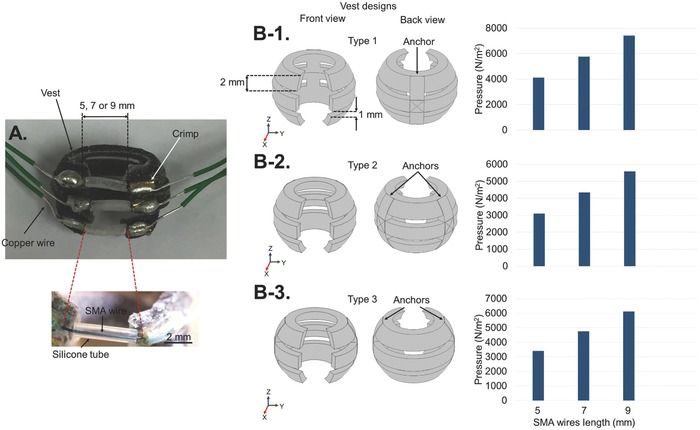
A) The fabricated Type 2 device with SMA wires. B‐1–B‐4) Schematics of the three designs for the vest, and their maximum positive pressure under 4.5% strain of the SMA wires at lengths of 5, 7, and 9 mm.

The SMA‐based device is composed of three SMA wires with brass crimps on both ends glued to a flexible vest. The SMA wires were then covered with silicone tubing. **Figure** **2**A shows the assembled vest with 5 mm length SMA wires wrapped with silicone tubing. After applying voltage to the device, the SMA wires contracted, and consequently applied a force on the vest. The deformation of the vest compressed the balloon or bladder thus leading to voiding. The details of each part of the device are as follows:

**SMA wire**: Crimped Nitinol (NiTi) SMA wires with diameters of 200 µm and lengths, *L*, of 5, 7, and 9 mm were purchased from Dynalloy Inc. (Irvine, CA).^16^ The SMA wires were capable of contracting up to 4.5%, Δ*L*/*L*, by applying the appropriate voltage (Δ*L* is the change in the length of the wire).^16^ The transformation temperature at which the contraction of wires happens was based on prior shape training.^38^ In this paper, we used a transformation temperature of 70 °C.^16^ Additional properties of the SMA material are described in Section 1 of the Supporting Information. The SMA wires we used exhibited a resistivity of 29 Ω m^−1^,^16^ (i.e., 0.145 Ω for 5 mm SMA wire). However, after the crimping and soldering, the resistance increased to about 0.7 Ω.

**Figure 3:**
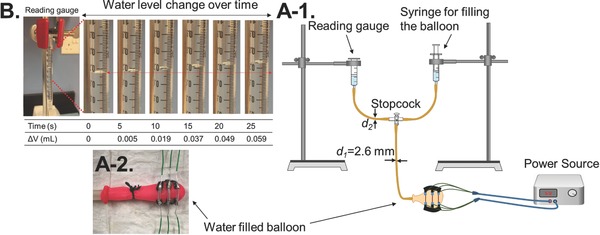
A‐1) The experimental setup for measuring the SMA wire devices. A‐2) The Type 3 device with 9 mm SMA wires wrapped around a balloon filled with water. B) The reading gauge representing the water voided over time for the Type 3 device with 9 mm SMA wires.

**Vest**: The spherical vest, which had an inner diameter of 1 cm and thickness of 1 mm, consisted of three rings that were connected at one or two anchors. The size of the vest was designed to match according to the normal size of a rat's bladder. The rings had a width of 2 mm to accommodate the brass crimps, and gaps of 1 mm between them. The open top and bottom for the vest, as well as the separate rings, simplified the application of the device onto the bladder. It also allowed monitoring of the surface of the bladder while actuating the device. The rings had 50° opening that were completed by the SMA wires to wrap completely around the bladder. Figure 2B‐1–B‐3 shows the three designs that were considered for the flexible vest. In these designs, the positions of the anchors, which restrained the deformation of the rings, were changed. The first design was named Type 1, where the rings were connected to each other at only one anchor. This created two equal sized ring sections (Figure 2B‐1). The second design was named Type 2, where the two anchors on opposite sides were positioned with a separation angle of 104° to each other. This created three approximately equal sized ring sections in the back and in the front (Figure 2B‐2). The third design was named Type 3, where the rings were connected by two anchors on opposite sides with a separation angle of 180°. This created one large ring section in the back of the structure, and two shorter ring sections in the front (Figure 2B‐3). The vests were designed in SOLIDWORKS and 3D printed by using a flexible rubber‐like material (i.e., TangoBlackPlus) with a Young's modulus, *E*, of 0.3 MPa,^39^ and a density of 1130 kg m^−3^.^40^ The flexibility of the vest allows the device to conform well to the bladder. It is important to note that the same configuration and dimensions for the rings can be used for a larger sized bladder by simply increasing the number of rings in order to cover the whole bladder surface.

**Silicone tubing**: Due to the temperature increase in the SMA wires after applying voltage, a thermal insulation layer was required in order to reduce the heat transfer to the balloon, and more importantly, to the bladder when we subsequently test the device in animals. The low thermal conductivity of silicone polymer (i.e., 0.2 W m^−1^ K^−1^)^41^, ^42^ made it a suitable thermal insulation layer for the SMA wires. For this purpose, medical grade silicone tubing (Silcon) with inner diameters of 500 µm and outer diameters of 965 µm were used as the thermal insulation layers for the SMA wires.

Upon the contraction of the SMA wires due to the voltage application, the flexible vest will deform and compress the bladder. Therefore, a study on the pressure and displacement changes of the vest was necessary for optimizing the device. The three vest designs in Figure 2B‐1–B‐3 were prepared in SOLIDWORKS for 3D printing, but they were numerically modeled in COMSOL. We have not included the SMA wires in the numerical modeling. Instead, displacement loads were applied to the tip of each finger to model the effect of force imposed by the contraction of the SMA wires onto the vest. The displacement loads were calculated based on the maximum 4.5% contraction of the SMA wires at various lengths. A displacement load of 0.1125, 0.1575, and 0.2025 mm were applied to each finger for SMA wire lengths of 5, 7, and 9 mm, respectively, along the *y*‐axis. Figure 2B‐1–B‐3 also shows the pressure distribution of the three vest designs for 5, 7, and 9 mm wires. The maximum positive pressure in the vest is increased by increasing the length of wires in each design. Higher positive pressure is a sign of more deformation imposed by the wires onto the vest, thus applying more force onto the balloon or bladder. The 3D pressure distribution for all the devices are shown in Figure S1 (Supporting Information), where the black solid lines show the devices before deformation. The device with longer SMA wires shows more deformation and a larger maximum pressure in Figure S1 (Supporting Information). Displacement changes of the vest should be used to compare the vest designs for each length of SMA wire, as the differences in the pressure distribution for different SMA wire lengths were not immediately obvious. This effect is explained after conducting the benchtop measurements on the fabricated devices.

## 3 The Experimental Approach to Study the Potential of SMA‐Based Devices

**Figure** **3** shows the experimental setup to measure the amount of water voided from a balloon with a size similar to the bladder of a rat. As shown in Figure 3A‐1, the device was placed on a balloon (Figure 3A‐2), which was then filled with water using a syringe. A power source was used to apply voltage to the device, and the displaced volume of water, Δ*V*, was read by a syringe that had its plunger removed. Figure 3B shows changes in the water level in the syringe upon voltage application to a Type 3 device with 9 mm SMA wires. The tube connected to the balloon had an internal diameter, *d*_1_, of 2.6 mm, while the diameter of the tube exiting from the three‐way stopcock, *d*_2_, was varied from 2.6 to 0.5 mm. This was done to find out the effects of *d*_2_ on voiding (reported in Figure S2, Supporting Information), and to model the balloon voiding system more closely after the rat's urinary system. The typical urethra opening for a young rat is 1.44 mm in diameter at the bladder's neck, but this reduces to 0.088 mm at the external sphincter.^43^ However, the *d*_2_ value of 0.5 mm was the smallest dimension that provided a good seal and fit with the three‐way stopcock. The volume of the balloon, *V*, was 1 mL for all the devices, similar to the bladder of the rat.

Figure S2 (Supporting Information) plots changes in volume, Δ*V*/*V*, as a function of the voltage applied to the Type 2 device with 9 mm SMA wires while changing the *d*_2_ diameter. As Figure S2 (Supporting Information) shows the volume voided increased with larger voltages for all the *d*_2_ diameter values, however, a clear trend was not observed for each voltage by increasing *d*_2_.

**Figure 4:**
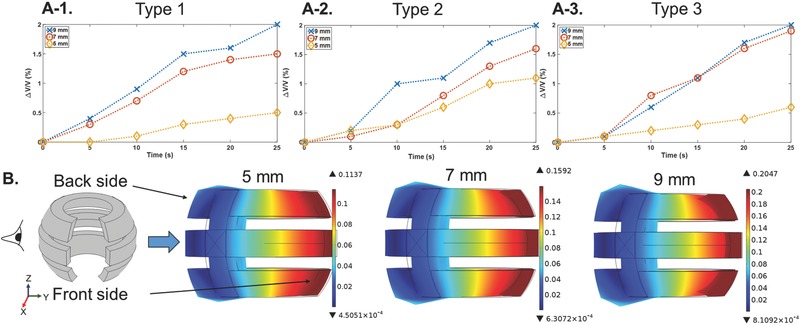
A‐1–A‐3) Comparison of measured Δ*V*/*V* versus time characteristics with *d*_2_ = 1.6 mm at 3 V for Type 1, Type 2, and Type 3 designs with 5, 7, and 9 mm SMA wires. B) Comparison of simulation results for the displacement of the Type 2 design under maximum strain change (side views).

The three vest designs were integrated with 5, 7, and 9 mm SMA wires, and measured as shown in **Figure** **4**A‐1–A‐3. A voltage of 3 V was applied to the three designs for a duration of 25 s. Δ*V*/*V* is plotted against time for the devices in Figure 4A‐1–A‐3. By increasing the length of the SMA wires, larger contractions occurred, which exerted larger forces on the vest, and resulted in larger values for Δ*V*/*V*. The simulation results for the displacement of the Type 2 device with 5, 7, and 9 mm wires under maximum strain are shown as an example in Figure 4B. By increasing the wire length, a larger displacement was observed for the vest, which was consistent with the experimental results for the device shown in Figure 4A‐2. A similar trend was observed for the simulation results of the Type 1 and Type 3 designs. Figure S3 (Supporting Information) shows the side views of the displacement changes in the three vest designs when the SMA wires of various lengths were under maximum strain. The maximum displacement in Figure S3 (Supporting Information) was increased by increasing the length of the SMA wires for each vest design, which is similar to the pressure changes in Figure S1 (Supporting Information).

**Figure 5:**
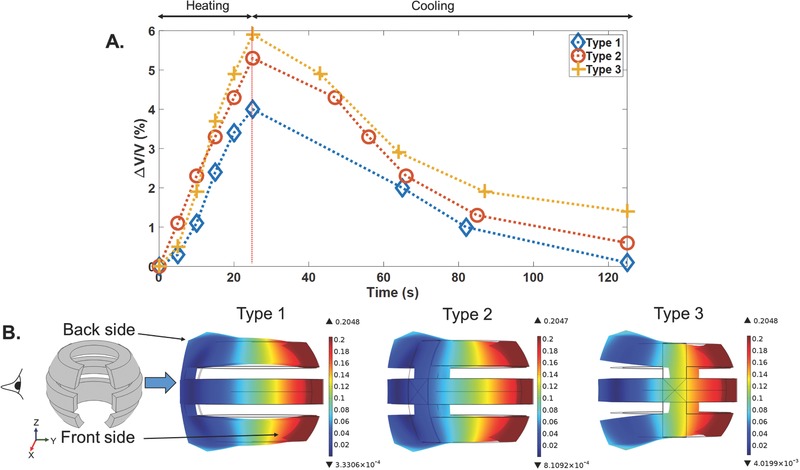
Type 1, Type 2, and Type 3 devices with 9 mm SMA wires. A) Plots of Δ*V*/*V* versus time, with *d*_2_ = 0.5 mm (the applied voltage of 5 V was turned off at 25 s). B) Comparison of the simulation results for the displacement under maximum strain change (side views).

Since 9 mm long SMA wires caused the largest deformation in the vests in Figure 4A‐1–A‐3, we characterized the changes in volume, Δ*V*/*V*, during and after voltage application in three vest designs with 9 mm SMA wires in **Figure** **5**A. The measurements were performed while applying 5 V for a duration of 25 s. At 25 s, we removed the voltage, which caused the wires to start to cool, and Δ*V*/*V* recovered to its original level. It is important to note that, by using the silicone tubing as the thermal insulator, the heat dissipation of the SMA wires will not be as quick as that found in bare SMA wires. The Type 3 device showed higher Δ*V*/*V* at 25 s in comparison with the Type 1 and Type 2 devices. Figure 5B shows the side views of the displacement changes in the three vests. As presented in Figure 5B, the back side of the Type 3 vest experienced larger deformations after the contraction in the SMA wires in comparison with the Type 1 and Type 2 designs. A similar effect was observed for the Type 3 vests with 5 and 7 mm SMA wires in Figure S3 (Supporting Information).

In order to find out the effect of voltage on Δ*V*/*V*, we applied voltages of 3, 4, and 5 V to the Type 3 device with 9 mm SMA wires, and the Δ*V*/*V* as a function of time is shown in Figure S4 (Supporting Information). A Δ*V*/*V* of 6% was obtained after applying 5 V for 25 s. Figure S4 (Supporting Information) shows that, by increasing the voltage, a larger value of Δ*V*/*V* was achieved. Similar to Figure 5A, after removing the voltage, the SMA wires tended to recover to their original lengths. It took a longer time for the Type 3 device to recover to its original Δ*V*/*V* level due to the higher temperatures induced in the wires. As shown in Figure S5A (Supporting Information), a 5 V voltage was applied to the Type 3 device for a duration of 55 s to find the maximum Δ*V*/*V* that could be achieved by this device. The value of ∆*V*/*V* was increased from 4.9% at *T* = 25 s to 7.1% at *T* = 55 s. The changes in the temperature of the SMA wires during 55 s of voltage application are shown in Figure S5B (Supporting Information). The temperature increased from 71.5 °C at *T* = 25 s to 76.5 °C at *T* = 55 s. Even the temperature only increased by 5 °C to 76.5 °C at *T* = 55 s and a higher level of voiding was achieved by continuing the voltage application, but it leads to overheating and starting to burn the SMA wires. To study the effect of larger voltage actuation on the shape memory property of the SMA wires, the change in volume, ∆*V*/*V*, was measured for twice voltage application to the device. The plot of ∆*V*/*V* versus time for two times of 5 V application is shown in Figure S5A (Supporting Information), and two times of 3 and 4 V applications are presented in Figure S5C (Supporting Information). The second time actuation of the device for 5 V resulted in a reduction of 1% for ∆*V*/*V* at *T* = 25 s, while this value is reduced to 0.4% and 0.2% at *T* = 25 s for 3 and 4 V applications, respectively.

**Figure 6:**
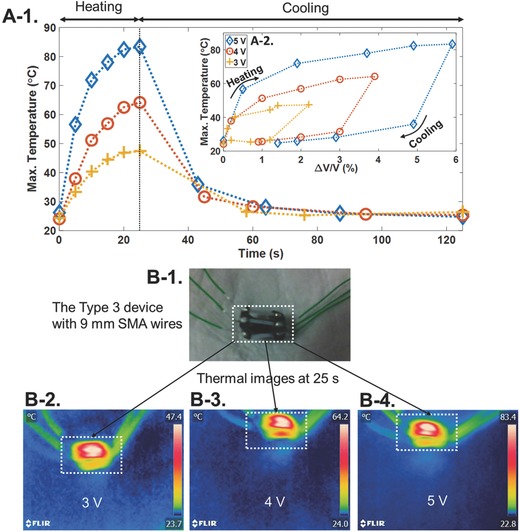
The Type 3 device with 9 mm SMA wires. A‐1) Plot of maximum temperature versus time. A‐2) Plot of maximum temperature versus water voiding percentage. B‐1) Optical and B‐1–B4) thermal images after applying different voltages for 25 s.

**Figure 7:**
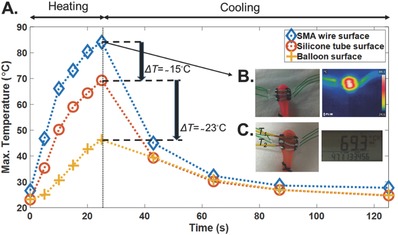
The Type 3 device with 9 mm SMA wires on the balloon. A) Plot of the maximum temperature versus time for the SMA wire surface (taken by the thermal camera), silicone tube surface, and the balloon surface (measured by the two‐channel thermometer). B) The optical and thermal images at 25 s after applying a voltage of 5 V. C) The connected thermocouples on the SMA wire and balloon surface, and the measured temperatures of channels (*T*_1_ and *T*_2_) at 25 s after applying a voltage of 5 V.

An FLIR E5 thermal imaging camera (Wilsonville, OR) was used to monitor changes in temperature on the Type 3 device with 9 mm SMA wires over time under 3, 4, and 5 V applied voltages. **Figure** **6**A‐1 shows the maximum temperature of the SMA wires as a function of time for the device. The voltage was removed at 25 s, thus the temperature started to decrease afterward. The device under 3 V voltage application in Figure 6A‐1 shows the lowest temperature change in the SMA wires. The maximum temperature versus Δ*V*/*V* is presented in Figure 6A‐2. The contraction of the device exhibits hysteresis, similar to the temperature‐strain changes in the SMA wires.^16^, ^38^ As shown in Figure 6A‐2, the device reached the maximum Δ*V*/*V* after 25 s of voltage application (heating of wires), after which Δ*V*/*V* started to decrease (cooling of wires). As reported in other work,^44^ we showed that, by increasing the voltage applied to the SMA wires, the induced temperature will increase. The device in Figure 6A‐2 showed Δ*V*/*V* of 6% for the 5 V application, while this value was reduced to about 2.5% for the 3 V application. More explanation on the SMA material properties, and temperature versus strain hysteresis behavior (Figure S6, Supporting Information) is given in the Supporting Information. Figure 6B‐1–B‐4 shows the optical and thermal images of the Type 3 device with 9 mm SMA wires at voltages of 3, 4, and 5 V for 25 s. The maximum temperature in the thermal images was found at the surface of the SMA wires, and it increased with increasing voltages. As these measurements were taken without the balloon, we also checked the temperature on the silicone tube and on the surface of a water filled balloon for the same device when 5 V was applied by using a two channel HH506RA thermometer (Stamford, CONN) in **Figure** **7**. One K‐type thermocouple was positioned on the surface of the silicone tube, and the other one was placed underneath the tube on the balloon surface. Kapton tape was used to fix the thermocouples in place, which might have caused the measured temperatures to be a bit higher than the real temperature values. Figure 7A plots the maximum temperature versus time for the SMA wire, the silicone tube, and the balloon surface. The optical and thermal images of the device under 5 V stimulation after 25 s is shown in Figure 7B. Figure 7C shows the positions of the thermocouples, and the temperatures of both channels at 25 s. The temperature reduction of about 15 °C between the surface of the SMA wire and the silicone tube was due to the thermal insulation of the silicone tube. A further 23 °C reduction in temperature was seen on the balloon surface due to the thermal conduction through the balloon surface and the air gap between the silicone tube and balloon surface. Since biological tissue has a thermal conductivity of 0.46 W m^−1^ K^−1^,^45^ the heat dissipation should happen faster than the rubber balloon.

## 4 In Vivo Animal Test of the Type 3 Device with 9 mm SMA Wires

**Figure 8:**
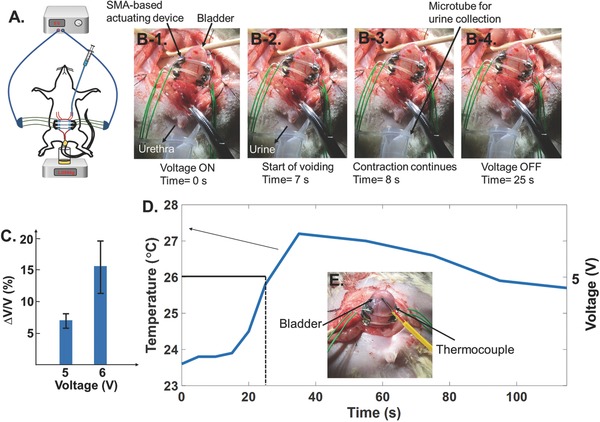
The Type 3 device with 9 mm SMA wires. A) The animal test setup, B‐1–B‐4) the voiding process during 25 s of 5 V voltage application, C) the voiding percentage for 5 and 6 V activations of the device, D) the temperature versus time curve for the 5 V actuation of the device during 25 s, E) the position of the thermocouple on the surface of the bladder.

The Type 3 device with 9 mm SMA wires was implanted onto the bladder of an anesthetized rat, as shown in **Figure** **8**A. The details of the surgical procedures are explained in the Supporting Information. The animal care and use procedures were approved by the Institutional Animal Care and Use Committee (IACUC) of the National University of Singapore. The methods were carried out in accordance with the R15‐0592 protocol. As shown in Figure 8A, normal saline was used to fill the bladder up to 0.7 mL via a catheter inserted in the ureter. A voltage of 5 V was applied to the SMA wire device for 25 s, and the urine voided during this time was collected and weighed. Considering a measured density of 1.019 kg L^−1^ for urine, a Δ*V*/*V* of 8.12% was achieved for the device. Figure 8B‐1–B‐4 shows the implanted device before and after the time that voiding started. This Δ*V*/*V* is quite different from the experimental results for this device shown in Figure 5A, which showed a Δ*V*/*V* of 6%. One reason for the difference was the fact that the water displacement from the balloon in the setup shown in Figure 4A‐1 was pushing against gravity into the reading gauge. For this reason, we also measured the voiding volume by disconnecting the outlet tube from the reading gauge in Figure 4A‐1, and letting the water be voided into a petri dish. This increased the voided amount to a Δ*V*/*V* of 7.7%. For comparison, the Young's modulus of the bladder muscle is at least two orders of magnitude smaller than that of the rubber balloon,^46^ thus a higher value of Δ*V*/*V* was not unexpected. However, the urethral resistance can affect the value of Δ*V*/*V*.^1^ By increasing the applied voltage to 6 V, a Δ*V*/*V* of 19.72% was measured, but in order to keep the temperature change in the SMA wires nearer to the animal's body temperature,^47^ and to also avoid overheating and thus shortening the lifetime for the device, a maximum voltage of 5 V will be used for the future implantations of the device. The 5 and 6 V activations of the device were repeated twice and Figure 8C presents the percentage of voiding for these activations. The voiding percentage was reduced from 8.12% for the first 5 V actuation to 5.76% for the second actuation. However, this reduction is from 19.72% to 11.31% for the first and second 6 V activations, respectively. This is consistent with the observations in Figure S5A,C (Supporting Information) that showed a larger reduction in Δ*V*/*V* for the second time actuation at a higher level of voltage. A K‐type thermocouple was used to measure changes in temperature for the surface of the bladder during 25 s of 5 V activation. Figure 8D shows the plot of temperature and voltage versus time for the device. An increase of about 3 °C was observed from *T* = 0 s to *T* = 35 s on the surface of the bladder. The position of the thermocouple on the bladder is shown in Figure 8E. Video S1 (Supporting Information) presents the bladder voiding under 5 V application for 25 s using the Type 3 device with 9 mm SMA wires. With additional design changes in the vest to improve the conformability of the vest after each cycle of actuation, it might be possible to utilize multiple cycles of the actuation to increase further the change in volume.

With 5 V stimulation, the current delivered through the device was measured at 2.48 A. Since the voiding started and completed after about 8 s of applying 5 V to the device (Video S1, Supporting Information), the time duration can be reduced from 25 to 8 s. Therefore, the temperature on the surface will be reduced to about 23.8 °C (Figure 8D). Furthermore, if we consider the need to void the bladder 11 times in 1 h for a rat,^48^ the energy consumption will be ≈60.016 mAh. Therefore, a normal 5 V, 5600 mAh battery will be sufficient to power the device for about 93 h. As an alternative, integrating energy harvesting mechanisms with implantable biomedical devices to create self‐sustaining biomedical systems is fast becoming a feasible option.^49^, ^50^ As an example, flexible energy harvesters have been shown to be capable of scavenging energy from organ motion to power integrated sensors.^51^, ^52^, ^53^ As a result, future integration of the proposed flexible actuator device with energy harvesters may enable the creation of a self‐sustaining bladder control system to void the bladder that has no batteries at all.

## 5 Discussion

The actuation time for the proposed SMA‐based devices was limited to 25 s for the maximum voltage of 5 V for bench top experiments, even though a longer time actuation improved the voiding percentage. This was due to the fact that the SMA wires were damaged by overheating during a longer stimulation. Even the overheating of SMA wires for a longer time may damage the wires and their contraction property, but no obvious degradation was observed for the silicone tube and vest after a few consecutive activation cycles of the device. The voiding of water from balloon started as soon as the voltage was turned ON and continued till the voltage was removed. In contrary to the balloon experiments the in vivo actuation of the device on bladder started the voiding after about 7 s of applying voltage and the voiding completed within 1 s. Even the voltage application continued afterward till *T* = 25 s, the force due to the gradual contraction of the SMA wires was not as large as the contraction force exerted after turning the voltage ON. As a result, no further voiding was observed by continuing the voltage application beyond 8 s. On the other hand, it was observed in Figure S5A,C (Supporting Information) that a shorter duration for applying voltage (i.e., 8 s) reduced the weakening of the shape memory property of the SMA wires after two times actuation of the device. Therefore, for the real implantation of the device on the bladder, the activation of the device can be done in several consecutive 8 s cycles for achieving higher voiding percentage and without being worried in shortening the device lifetime. A future improvement in the voiding percentage of the device is considered to further reduce the number of activation cycles for achieving a complete voiding in a shorter time.

## 6 Conclusion

We have demonstrated for the first time a new flexible 3D printed medical device driven by SMA actuators that can be used to void the bladder. The device does this by physically contracting the bladder using the forces exerted by stimulated SMA wires. The device was designed, fabricated, and experimentally tested in various configurations in order to optimize the design for animal testing. We also studied the temperature changes around the device to ensure that the heating generated by the SMA wires will not damage the bladder and the surrounding tissue. The in vivo test of the optimized device on a rat bladder showed a voiding volume of 8% in one cycle of voltage application. Our results demonstrate that this type of device can potentially be used in patients that are unable to void their bladder due to a variety of clinical conditions.

## Conflict of Interest

The authors declare no conflict of interest.

## Supporting information

Supplementary

Supplementary
