# Supplementary material for: A 3D Printed Implantable Device for Voiding the Bladder Using Shape Memory Alloy (SMA) Actuators
Source: Adv Sci (Weinh). 2017 Jul 26;4(11):1700143. doi: 10.1002/advs.201700143 (PMC5700638; doi:10.1002/advs.201700143)
Supplement: Supplementary file 1 — Supplementary [file ADVS-4-na-s001.pdf]

Copyright WILEY-VCH Verlag GmbH & Co. KGaA, 69469 Weinheim, Germany, 2017.

ADVANCED SCIENCE
Open Access

Supporting Information

for *Adv. Sci.,* DOI: 10.1002/advs.201700143

A 3D Printed Implantable Device for Voiding the Bladder Using Shape Memory Alloy (SMA) Actuators

Faezeh Arab Hassani, Wendy Yen Xian Peh, Gil Gerald Lasam Gammad, Roshini Priya Mogan, Tze Kiat Ng, Tricia Li Chuen Kuo, Lay Guat Ng, Percy Luu, Shih-Cheng Yen, and Chengkuo Lee**

Copyright WILEY-VCH Verlag GmbH & Co. KGaA, 69469 Weinheim, Germany, 2016.

Supporting Information

A Three-Dimensionally (3D) Printed Implantable Device for Voiding the Bladder using Shape Memory Alloy (SMA) Actuators

Faezeh Arab Hassani, Wendy Yen Xian Peh, Gil Gerald Lasam Gammad, Roshini Priya Mogan, Tze Kiat Ng, Tricia Li Chuen Kuo, Lay Guat Ng, Percy Luu, Shih-Cheng Yen, and Chengkuo Lee

Table of Content

Supplemental Section 1: Shape memory alloy materials…………………………….. 2

Supplemental Section 2: Surgical procedure………………………………………….. 2

References……………………………………………………………………...… 4

Supplemental Figure 1………………………………………………………………….. 5

Supplemental Figure 2………………………………………………………………….. 6

Supplemental Figure 3………………………………...…………………………….. 7

Supplemental Figure 4………………………………………………………………….. 8

Supplemental Figure 5………………………………………………………………….. 9

Supplemental Figure 6…………………………………………….....…………….. 10

Supplemental Video 1 (Attached as a separate file)…………………………………... 11

Supplemental Section 1: Shape memory alloy materials

Shape memory is a property of some materials that allows them to remember their original shape. The shape memory property is due to atomic-level structural changes in the material from the martensitic to the austenite phase by applying heat.[1] The transition temperature of shape memory alloys such as Nickel Titanium (Nitinol) can vary within a range of -50 ^{o}C to 110 ^{o}C.[2] The original thermo-mechanical processing of the shape memory alloy wires defines the transition temperature.[3] If an alloy in a fully martensitic phase is heated, the austenite phase starts at the austenite start temperature, A_s, and the transformation is complete at the austenite finish temperature, A_f. When the cooling of the alloy in a fully austenite phase is started, the martensitic phase starts at the martensitic start temperature, M_s, and finishes at the martensitic finish temperature, M_f. The temperature versus strain for a SMA wire is presented in Supplemental Figure 1.[4] The SMA wires used in our device, has a A_s temperature of 70 ^{o}C, which causes their length to reduce with heat. In this case, the temperature change was caused by the voltage application. We have chosen 200 μm as the minimum diameter for the SMA wires since the thinner wires did not show enough force to deform the vest and pressurize the balloon. The biocompatibility of Nitinol also makes it a suitable material for medical implants.[4]

Supplemental Section 2: Surgical Procedure

The experiments were performed on an adult female Sprague Dawley rat (320 g). The animal care and use procedures were approved by the Institutional Animal Care and Use Committee (IACUC) of the National University of Singapore. The methods were carried out in accordance with the R15-0592 protocol. The animal was anaesthetized by injecting ketamine-xylazine (75mg/kg-10mg/kg) into the intraperitoneal space (IP), and checked for the depth of anaesthesia. Carprofen (5 mg/kg) and normal saline (0.2-0.5 mL/10g) were injected subcutaneously (SC) to provide analgesic and prevent dehydration of the animal. The hair on

the abdominal/pelvic area was shaved prior to making an incision. Ophthalmic ointment was placed on both eyes, and the body temperature of the rat was maintained with water recirculating heat pads.

Skin incisions were made at the lower abdominal area for positioning the device within the animal. To access the urinary bladder and left ureter, incisions of the subcutaneous tissue, muscle, and peritoneum beneath the skin on the ventral abdominal/pelvic region were made. Fat and connective tissue were carefully dissected away or gently pushed aside until the targeted tissues were visually identifiable and accessible. Micro-retractors were used to gently open the surgical field and to retract other organs, with great care taken to avoid stretching the vascular bundle or damaging them. A micro cannula was inserted into the left ureter for injecting normal saline to dilate the urinary bladder before the device actuation. The SMA wire device was positioned on the bladder wall and connected to the power source. The wires and catheter were held in place or anchored with sutures to neighbouring tissues such as muscles.

References

[1] L. An, W.M. Huang, *Mat. Sci. Eng. A* **2006**, *420*, 220.

[2] S. Barbarino, E. I. Saavedra Flores, R. M. Ajaj, I. Dayyani, M I Friswell, *Smart Mater. Struct.* **2014**, *23*, 1.

[3] K. Wada, Yong Liu, *Mat. Sci. Eng. A* **2008**, *481–482*, 166.

[4] DYNALLOY, Inc., Technical Characteristics of FLEXINOL Actuator Wires, www.dynalloy.com/pdfs/TCF1140.pdf, accessed: March, 2017.

[5] M. Morita, T. Hashimoto, K. Yamauchi, Y. Suto, T. Homma, Y. Kimura, *Materials Transactions* **2007**, *48*, 352.

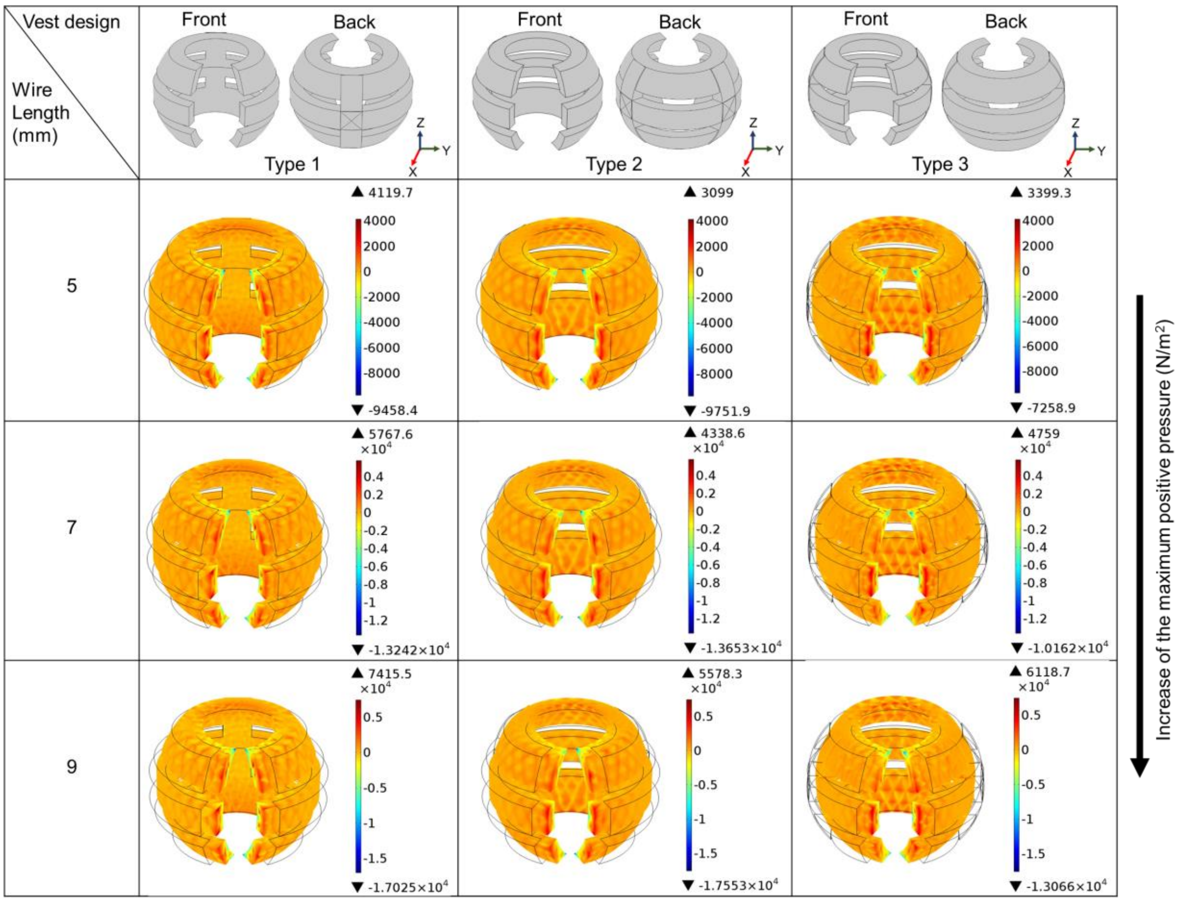

Supplemental Figure 1: The 3D simulation results for the pressure distribution of the Type 1, Type 2, and Type 3 vest designs, integrated with various SMA wire lengths.

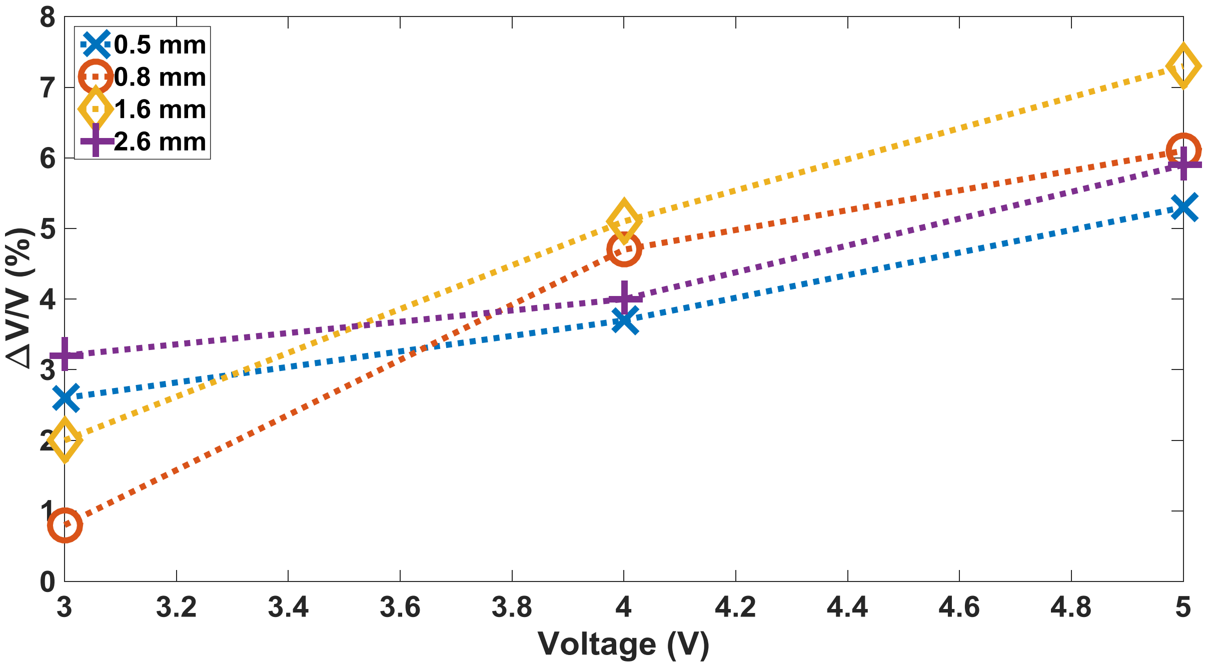

Supplemental Figure 2: The effect of the d_2 dimension on the volume voided at various applied voltages for the Type 2 device with 9 mm SMA wires.

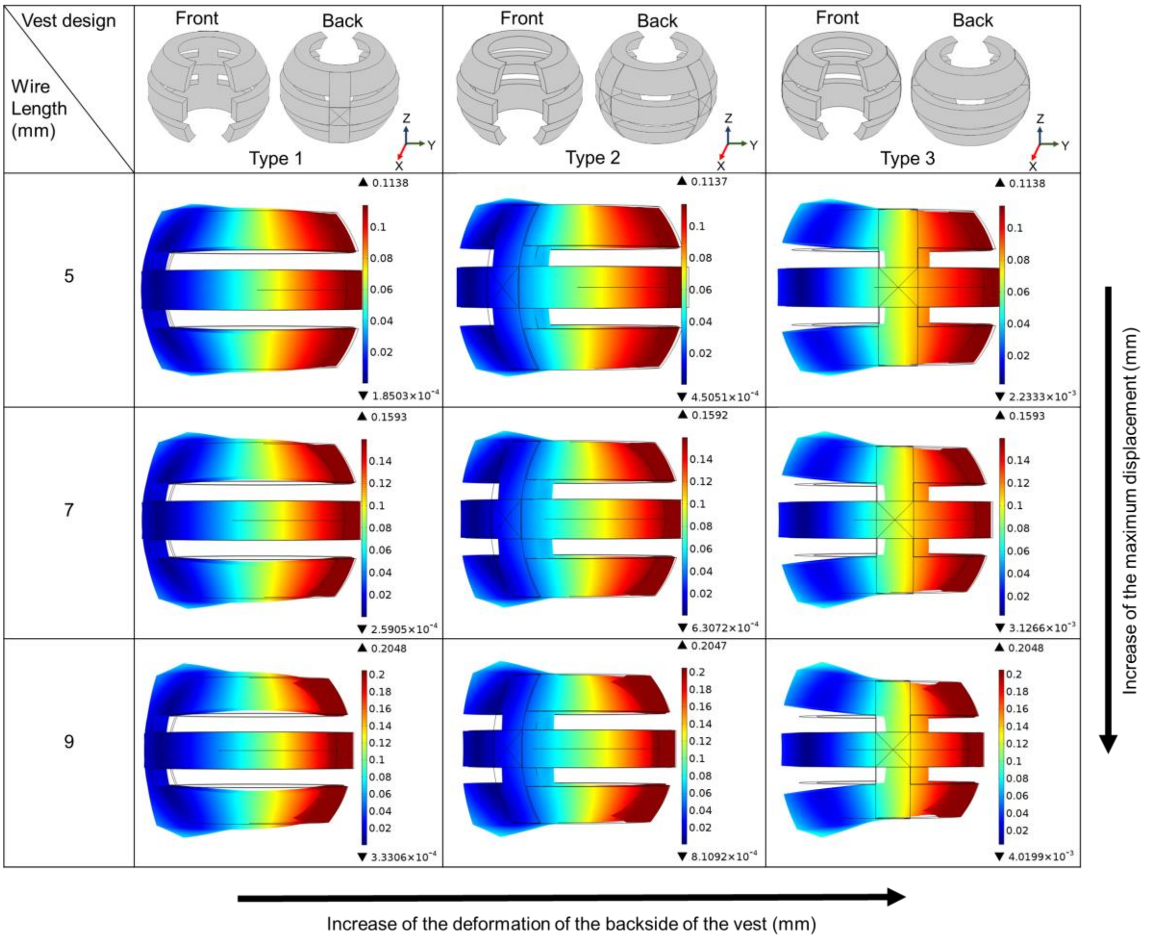

Supplemental Figure 3: The side view of the 3D simulation results for the displacement of the Type 1, Type 2, and Type 3 vest designs, integrated with various SMA wire lengths.

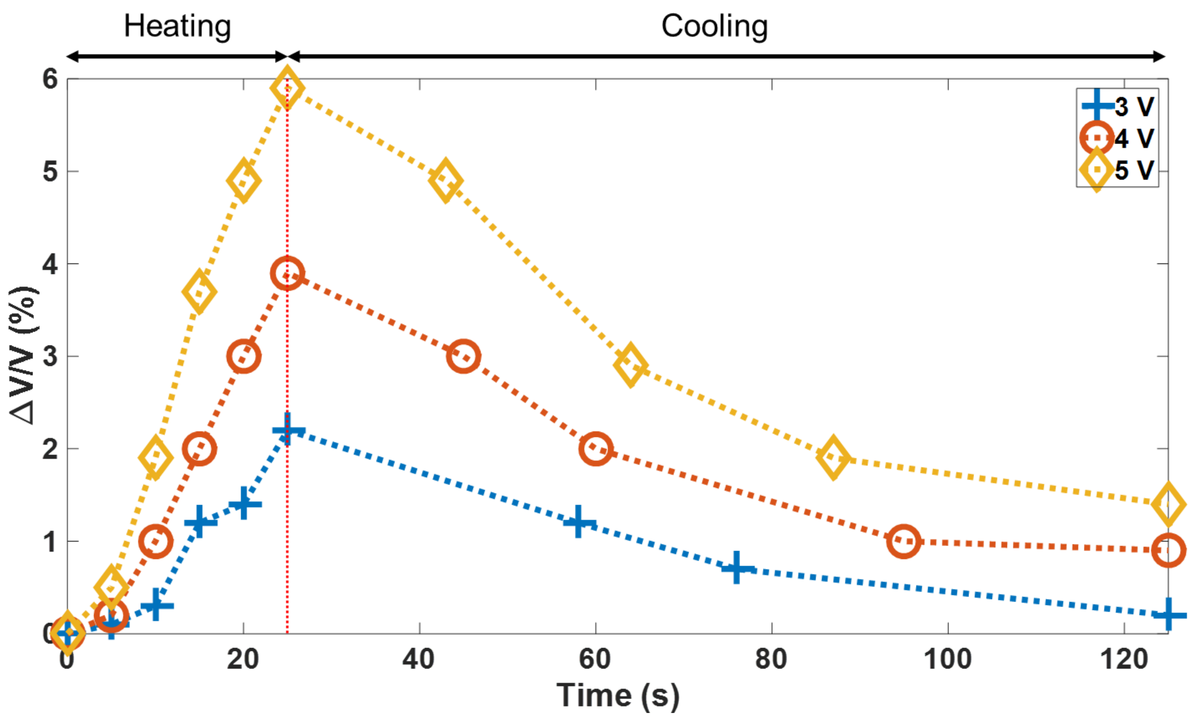

Supplemental Figure 4: The effects of voltage increase on voiding for the Type 3 device with 9 mm SMA wires with d_2= 0.5 mm.

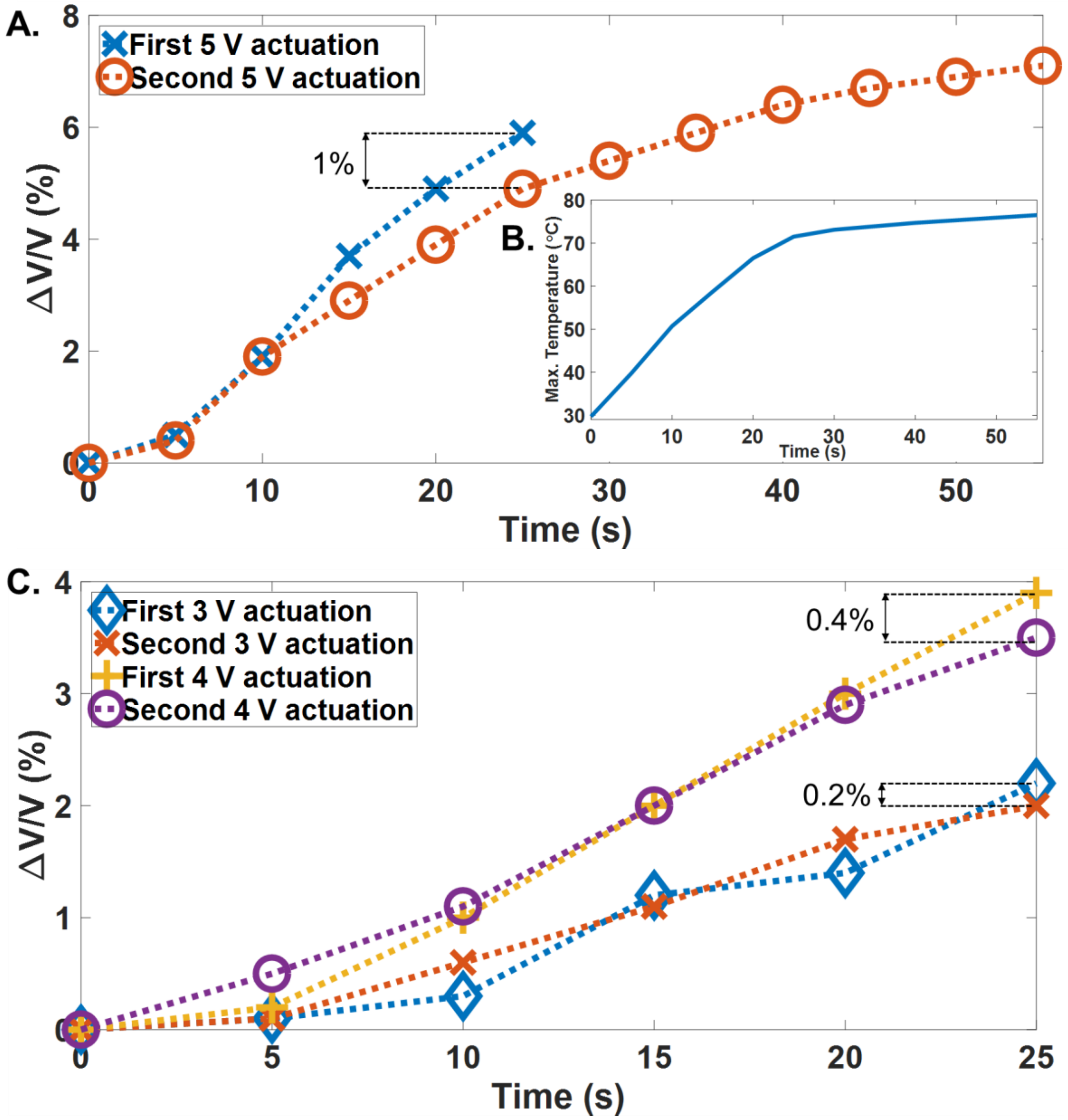

Supplemental Figure 5: The Type 3 device with 9 mm SMA wires with d_2= 0.5 mm. A) The plot of $\Delta V/V$ versus time when applying a voltage of 5 V for first 25 s, and second 55 s activations. B) Temperature versus time for 55 s of 5 V actuation. C) The plot of $\Delta V/V$ versus time when applying a voltage of 3 and 4 V for two times during 25 s.

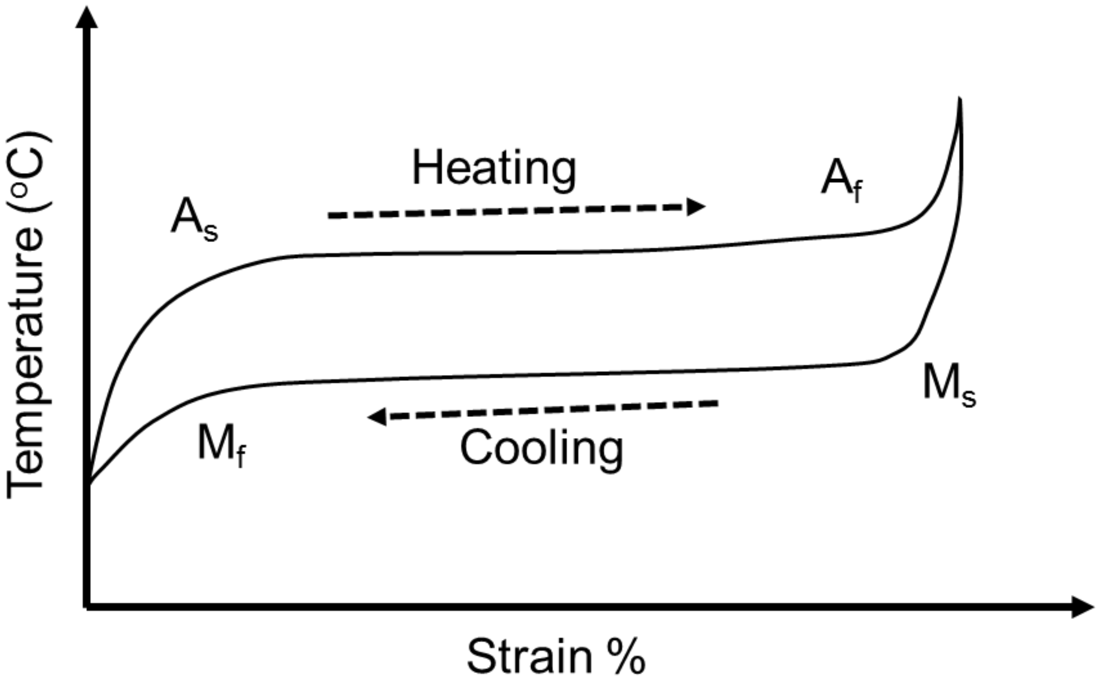

Supplemental Figure 6: Plot of the temperature versus strain of a SMA wire.

(Attached as a separate file)

Supplemental Video 1. The voiding process during 25 s of 5 V voltage application to Type 3 device with 9 mm SMA wires.
