# Supplementary material for: A 3D Printed Implantable Device for Voiding the Bladder Using Shape Memory Alloy (SMA) Actuators
Source: Adv Sci (Weinh). 2017 Jul 26;4(11):1700143. doi: 10.1002/advs.201700143 (PMC5700638; doi:10.1002/advs.201700143)
Supplement: Supplementary file 2 — Supplementary [file ADVS-4-na-s002.pptx]

## Slide 1
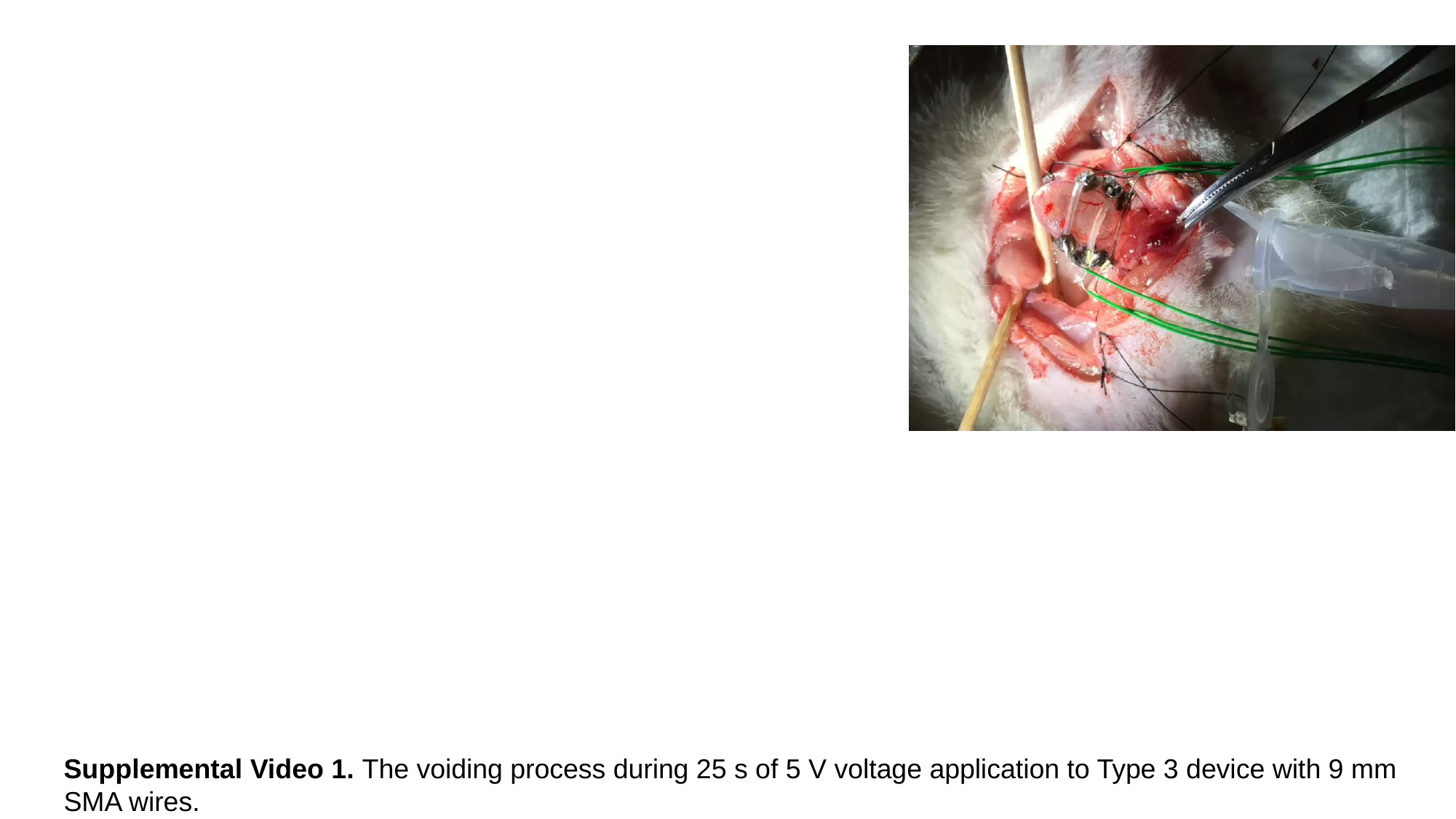

Supplemental Video 1. The voiding process during 25 s of 5 V voltage application to Type 3 device with 9 mm SMA wires.
